# The Effect of Artificial Insemination and Multiple Ovulation Embryo Transfer on Production, Health Status, and Survival of Holstein–Friesian Cows

**DOI:** 10.3390/vetsci13040320

**Published:** 2026-03-26

**Authors:** Eszter Tóth, János Posta, István Komlósi, Zoltán Szelényi, Michael Gonda, József Rátky, Renáta Knop

**Affiliations:** 1 Doctoral School of Animal Science, University of Debrecen, 4032 Debrecen, Hungary; 2 Department of Animal Science, Institute of Animal Science, Biotechnology and Nature Conservation, Faculty of Agricultural and Food Sciences and Environmental Management, University of Debrecen, 4032 Debrecen, Hungary; komlosi@agr.unideb.hu (I.K.); dr.knop.renata@agr.unideb.hu (R.K.); 3 Department of Obstetrics and Farm Animal Medicine Clinic, University of Veterinary Science, István Street 2, 1078 Budapest, Hungary; szelenyi.zoltan@univet.hu (Z.S.);; 4 Department of Animal Science, South Dakota State University, Animal Science Complex, Box 2170, Brookings, SD 57007, USA; michael.gonda@sdstate.edu

**Keywords:** Holstein–Friesian, multiple ovulation embryo transfer, artificial insemination, survival, health status

## Abstract

The effects of reproductive technologies, namely, artificial insemination (AI) and multiple ovulation embryo transfer (MOET), on the production, health, and longevity of Holstein–Friesian cows were evaluated. Data were obtained from the time period between 2017 and 2024 on a Hungarian dairy farm and consisted of 1783 cows (1544 AI and 239 MOET). Deep-frozen semen from identical bulls was used for both the AI and MOET groups. Disease incidence, productive life, and early-lactation milk production phenotypes were collected in these cows. MOET cows demonstrated significantly higher milk yield during the first 100 days of lactation but had a 43.9% greater risk of culling compared with AI cows (*p* < 0.05). Metabolic and reproductive disorders were the most common reasons for culling cows, with increased frequency of health issues correlating with higher culling risk ratios (*p* < 0.05). While MOET cows showed lower incidences of metabolic disorders, reproductive problems, and mastitis, their shorter productive lifespan likely limited overall disease exposure. Nevertheless, when MOET cows experienced illness, the impact was more severe, particularly in relation to metabolic issues (*p* < 0.05). These findings highlight trade-offs between improved genetic potential and health resilience in MOET-derived cows. Despite their higher productivity, their management may require greater health vigilance. This study offers practical insights for dairy producers in selecting reproductive strategies to balance genetic gain, herd health, and longevity under intensive production systems.

## Simple Summary

This study compared milk production, health, and longevity traits between Holstein–Friesian cows produced using artificial insemination (AI) and multiple ovulation embryo transfer (MOET) on a Hungarian dairy farm from 2017 to 2024. Records from 1783 cows (1544 AI; 239 MOET) were analyzed for disease occurrence, culling risk, and early-lactation milk yield. The MOET-derived cows produced more milk during the first 100 days of lactation but had a 43.9% higher culling risk than AI cows. Although MOET cows had fewer cases of metabolic and reproductive disorders and mastitis, their shorter productive life may have limited disease exposure. Health issues, when they occurred, were more severe in MOET cows, especially for metabolic disorders. These results highlight a trade-off between higher genetic potential and reduced health resilience in MOET-derived cows, suggesting the need for stronger health management strategies despite their productivity advantages.

## 1. Introduction

The factors affecting the lifespan of Holstein dairy cows have been investigated in several studies. The productive lifespan refers to the period during which a cow is producing milk in the herd. In modern dairy farming, culling due to old age is uncommon, as economic considerations demand maintaining consistently high milk production, regular calving, and good overall health, all of which influence farmers’ decisions regarding optimal culling time [1]. Higher milk yield has been associated with an increased risk of culling, particularly when accompanied by elevated somatic cell counts and advancing age [2]. Additionally, conformation traits are also related to productive lifespan, with larger-framed cows often exhibiting longer productive lives [3].

Holstein cows differ from other breeds in both lifespan and lifetime milk yield [4]. In European Holstein populations, the productive lifespan of cows in commercial dairies has declined by approximately one lactation. Paternal inheritance showed the strongest influence on longevity and lifetime production traits, followed by lineage or related group, common Holstein ancestry, and breed effects [4]. These findings suggest that selective breeding for longevity and lifetime production traits can advance dairy cattle breeding programs.

Production diseases represent a major constraint on dairy cow longevity. Common health challenges include reproductive disorders, mastitis, and metabolic imbalances [5]. Key indicators of disease often include reduced milk yield, weight loss, low body condition scores, and behavioral changes [5]. Changes in body energy reserves during the dry period have been shown to predict postpartum health, with cows suffering reproductive tract disorders experiencing greater negative energy balance than healthy cows [6]. These results highlight the importance of monitoring health during both lactation and the dry period. Grzesiak et al. [7] reported low survival probability in cows affected by metabolic, digestive and respiratory diseases.

Holstein cows are susceptible to various postpartum diseases that impair productivity and reproductive performance. Early-lactation clinical disease signs include higher β-hydroxybutyrate levels and lower milk yield [8]. Common health disorders such as milk fever, retained placenta, metritis, ovarian cysts, mastitis, and lameness negatively influence reproductive parameters, including days to first insemination and number of services per conception [3]. Prepartum behavioral changes may serve as early indicators of postpartum diseases [9]. However, calfhood diseases are not reliable predictors of the later health status or first lactation performance of cows [10]. Continuous health monitoring is, therefore, crucial for improving management and sustaining productivity.

The increasing incidence of production-related disorders parallels the intensification of milk production on farms. In addition, embryo transfer (ET), including multiple ovulation embryo transfer (MOET) technology, has become an important tool in cattle breeding, enabling accelerated genetic improvement and offspring production from superior females [11]. Comparative studies have shown that heifers born via MOET exhibit lower pre-weaning mortality, improved reproductive performance during first lactation, and higher abundance of fertility-related transcripts compared with AI-derived heifers [12]. ET also facilitates international genetic exchange and represents a cost-effective alternative to live animal transport [11,13]. The ET industry has grown substantially, with more than 1.5 million transferable bovine embryos collected in 2018 [14]. Despite the extensive phenotyping in the dairy industry, data on the long-term effects of assisted-reproduction technologies (ARTs) on cow health, productivity and longevity are surprisingly limited. Modern high-throughput phenotyping systems have generated large datasets at both individual and herd levels [15,16].

The increasing demand for rapid adaptation to changing environments and increasing emphasis on animal welfare and disease resistance have accelerated the adoption of genomic selection (GS) in dairy breeding programs. GS strengthens modern breeding programs by using genomic data to predict breeding values and rank selection candidates [17,18]. When GS is combined with assisted-reproduction technologies, genetic gain can be further accelerated through reduced generation intervals [19].

ET can also be used to obtain offspring from elite cows with fertility problems [20]. Since the best genetic individuals often serve as donors, many ET-derived offspring already inherit superior genetics, increasing robustness and milk production. MOET-derived heifers were less likely to be culled during their first year of life and showed improved reproductive performance during their first lactation compared with AI-derived heifers [12]. However, ET-derived cows do not consistently outperform AI-derived cows across all economically relevant traits. For example, Lafontaine et al. [21] reported a longer interval from first service to conception for ET cows compared with AI cows.

Our research aimed to test the hypothesis that MOET-derived cows had higher first lactation milk production, lower incidence of reproductive and health disorders, and longer productive lifespan compared with AI-derived cows. Because MOET programs are typically applied to genetically superior donor animals, potential differences between AI- and MOET-derived cows may partly reflect inherited genetic merit in addition to reproductive technology.

## 2. Materials and Methods

Data were collected from a single Holstein–Friesian dairy herd at a cattle farm located in south-eastern Hungary. The study was conducted using farm data only and did not involve any animal experiments; therefore, ethical approval was not required. The data collection covered the period from 2017 to 2024. During the data collection period, the herd increased from 620 cows in 2017 to 850 in 2024, with 600–700 replacement heifers. All animals were housed in loose housing systems, with heifers in deep-bedded barns and lactating cows in free-stall barns. The facilities provided well-ventilated, pavilion-style structures; ad libitum access to water and total mixed ration (TMR) adjusted for age and physiological status; and automated feed delivery and manure removal. Milking was performed twice daily in a 40-stall carousel parlor, and environmental control (fans and sprinklers) mitigated heat stress during the summer months. Records on 1783 cows were used, of which 1544 cows were conceived via artificial insemination (AI) and 239 via multiple ovulation embryo transfer (MOET). Deep-frozen semen from identical bulls was used for both the AI and MOET groups to minimize paternal variation between the groups. The MOET procedure was performed exclusively on heifers, and embryos were produced locally within the same herd rather than imported from external breeding populations. Consequently, both AI- and MOET-derived animals originated from a comparable genetic background and were raised under identical management conditions through the study period. Birthdates and the date of culling (if the cow was culled) were recorded, along with the following records:

- Method of conception: MOET or AI;
- Metabolic disease (ketosis, hypocalcemia, fatty liver, and subacute ruminal acidosis) records: two levels (0 = no problem; 1 = at least one problem occurred during lifetime);
- Hoof disorder (digital dermatitis, interdigital phlegmon, sole ulcers, and laminitis) records: five levels (0 = no problem; 1–4 = number of problems during lifetime, with 4 indicating four or more);
- Uterine and reproductive disorder (metritis, clinical endometritis, retained fetal membranes, pyometra, cystic ovarian disease, anestrus, and uterine prolapse) records: five levels (0 = no problem; 1–4 = number of problems during lifetime, with 4 indicating four or more);
- Udder-related disorder (mastitis, udder edema, udder cleft dermatitis, and structural disorders) records: five levels (0 = no problem; 1–4 = number of problems during lifetime, with 4 indicating four or more).

The above-mentioned categorization was chosen to avoid sparse data structures and ensure stable parameter estimation in the statistical models. Given the relatively low frequency of higher-order health events, treating these variables as continuous or highly detailed categorical variables would have resulted in sparse cells and unreliable estimates. Grouping the data into ordinal categories (0, 1, 2, 3, ≥4) allowed for a biologically meaningful classification while maintaining sufficient sample size within each category, thereby improving the robustness and interpretability of the culling risk analysis. Health disorder diagnoses were performed by the veterinarian of the farm, and so were veterinary diagnoses. The distribution of health problems by conception method was compared using cross-tabulations in IBM SPSS Statistics 26.0. When cross-tabulations revealed a significant difference, post hoc analyses using adjusted standardized residuals were performed to identify the cells contributing to the overall association. The production data of all 1783 Holstein–Friesian cows were analyzed. Milk production during the first 100 days of lactation was analyzed as each cow was alive in this production stage. Homogeneity of variances was assessed with Levene’s test. The variances of the two groups were homogenous (F = 0.814; *p* > 0.05), and thus, the production data were compared between MOET and AI cows using an independent samples *t*-test in IBM SPSS Statistics 26.0. The dataset used for the survival analysis contained 53.017% cows that were still alive, which were marked as right-censored records. The number of calvings was used as a measure of longevity and treated as the dependent variable. The conception method was included as a time-independent variable. The various categories of health problems (metabolic, leg, reproductive, and udder-related) were included as time-dependent variables, whereas the first 100 days of lactation were included as a covariate in the model. The relationships between these factors and longevity were analyzed using the Weibull model in the Survival Kit v6.12 software [22]. The Weibull model was selected due to its flexibility in modeling time-dependent hazard functions commonly observed in longevity studies. The proportional hazards assumption inherent to the model was assessed based on graphical evaluation and model diagnostics, and no major deviations were detected. Model adequacy was evaluated using likelihood-based diagnostics, including convergence assessment and likelihood ratio statistics. The risk of culling was expressed using risk ratios, which represent the relative risk of a cow in a certain class to be culled compared with a cow in the reference class (risk ratio = 1).

## 3. Results

The culling reasons across the reproduction techniques are shown in Table 1. The culling reasons ‘accident’ and ‘other’ were excluded from the statistical analysis. The distributions of the remaining five culling reasons between the two reproduction techniques were compared with cross-tabulation, and a significant difference (χ^2^ = 9.893; *p* < 0.05) was found. The highest proportion of cows was culled due to reproductive disorders in each group. The frequency of a non-categorizable culling reason (other) was the lowest for both groups.

Uterine and reproductive problems were also the leading cause of culling (37.1%) in animals bred with MOET. However, a higher proportion of culling was due to metabolic diseases (15.5%) compared with the AI group, which was found to be significant based on adjusted standardized residuals (|z| = 2.5). Problems with hooves accounted for 18.1% of cases in this group, while udder-related problems (8.6%) and accidents (2.6%) had a lower rate. Culling for other reasons did not occur in this group.

**Figure 1:**
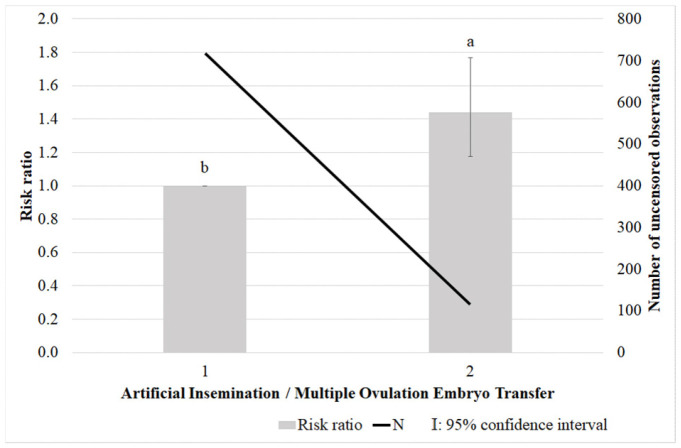
Comparison of culling risk (risk ratio (RR)) between cows born via artificial insemination (AI) and multiple ovulation embryo transfer (MOET). ^a,b^: the different letters show significant differences (*p* < 0.05).

The Weibull survival model converged properly, as indicated by the very small, standardized norm of the gradient of the −2 log-likelihood (1 × 10^−5^). The final model achieved a −2 log-likelihood value of 3876.51. Figure 1 illustrates the differences in culling risk between two groups of cows based on reproductive technology. Cows born through AI served as the reference group (RR = 1.0). The culling risk resulting from the survival analysis for cows born from MOET was higher (*p* < 0.05), with a risk ratio of 1.439, indicating a 43.9% increased likelihood of being culled compared with their AI-born counterparts.

In addition to identifying the primary causes of culling (i.e., metabolic disorders, lameness, reproductive disorders, and udder problems), we also assessed the incidence of these conditions between the two reproductive origin groups (AI vs. MOET).

**Figure 2:**
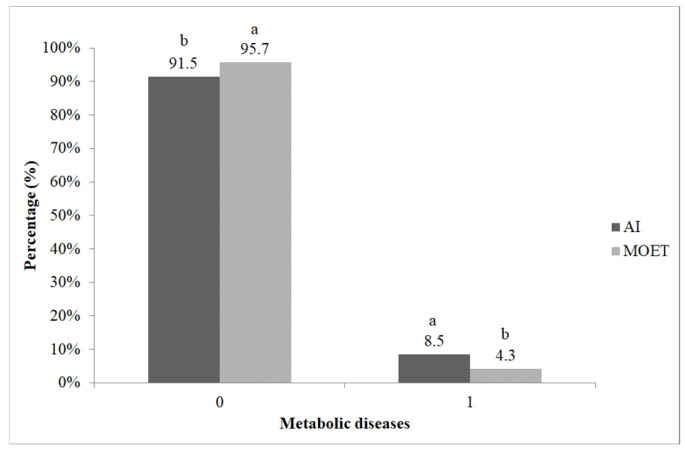
Incidence of metabolic disorders in cows born via artificial insemination (AI) and multiple ovulation embryo transfer (MOET). ^a,b^: the different letters show significant differences between groups within categories (*p* < 0.05).

As shown in Figure 2, the incidence of metabolic disorders was significantly lower (χ^2^ = 5.059; *p* < 0.05) in cows born via MOET compared with those born via AI. The adjusted standardized residuals (|z| = 2.2) confirmed that this difference contributed to the overall association, with MOET cows having fewer metabolic disorders than expected and AI cows having more.

**Figure 3:**
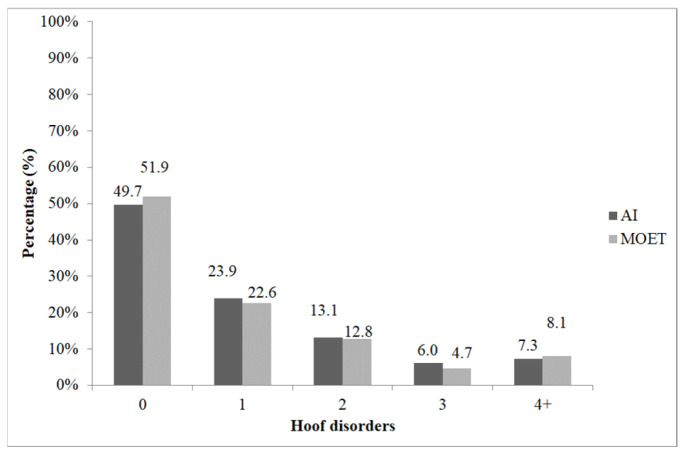
Incidence of hoof disorders in cows born from artificial insemination (AI) and multiple ovulation embryo transfer (MOET). 0: healthy individuals; 1: individuals diagnosed with lameness once; 2: cows diagnosed with lameness twice; 3: individuals that suffered from leg problems three times; 4+: cows affected four or more times.

Regarding leg disorders, 51.9% of the cows born via MOET showed no problems during their productive lifespan. Lameness was diagnosed in 22.6% of the animals, while 12.8% of them required treatment twice (Figure 3). Cows with three different occurrences of leg problems accounted for 4.7% of the group, and those affected more than four times represented 8.1%. There was no difference in the distribution of the occurrence of hoof disorders between the methods of conception (AI vs. MOET) (χ^2^ = 1.207; *p* > 0.05).

**Figure 4:**
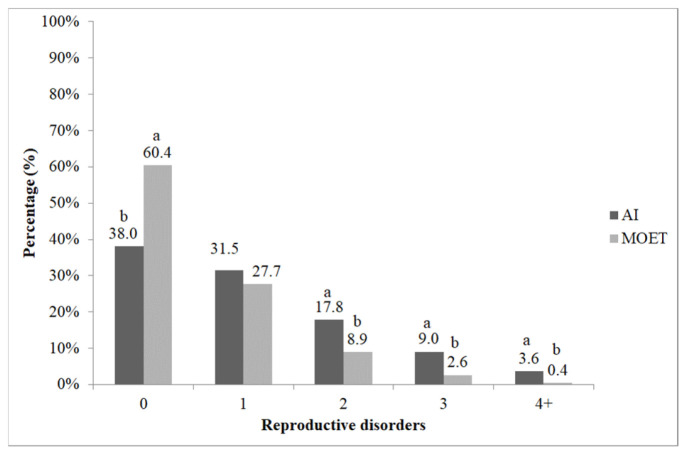
The proportion of reproductive disorders in cows born via artificial insemination (AI) and multiple ovulation embryo transfer (MOET). 0: healthy cows; 1: cows treated once; 2: cows treated twice; 3: cows treated three times; 4+: cows treated four or more times. ^a,b^: the different letters show significant differences between groups within categories (*p* < 0.05).

Regarding reproductive biological problems, the proportion of healthy individuals was lower among cows born using AI, comprising only 38% of that group (Figure 4). In contrast, 60% of cows born via MOET were free from reproductive issues. The frequency of reproductive problems was different (χ^2^ = 52.454; *p* < 0.05) in the MOET compared with the AI group. Post hoc analysis using adjusted standardized residuals indicated lower incidence of reproductive disorders for group 0 (|z| = 6.5), group 2 (|z| = 3.4), group 3 (|z| = 3.4) and group 4+ (|z| = 2.6), respectively.

**Figure 5:**
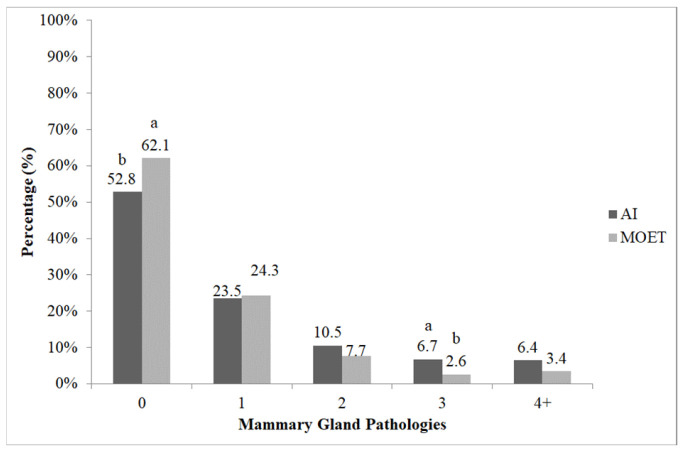
The proportion of mastitis cases among cows born via artificial insemination (AI) and multiple ovulation embryo transfer (MOET). 0: healthy cows; 1: cows treated once; 2: cows treated twice; 3: cows treated three times; 4+: cows treated four or more times. ^a,b^: the different letters show significant differences between groups within categories (*p* < 0.05).

Regarding the incidence of mastitis, a relationship was also found concerning the reproductive technique used. Cows born via MOET exhibited a lower incidence of mastitis compared with those born via AI, as cross-tabulation analyses showed significant difference between the two distributions (χ^2^ = 13.788; *p* < 0.05) (Figure 5). The proportions of healthy individuals in the MOET group were higher compared with the AI group, based on adjusted standardized residuals (|z| = 2.7). Adjusted standardized residuals (|z| = 2.5) also indicated a lower incidence of mammary gland disorders in MOET cows within group 3.

Milk yield variances between MOET- and AI-derived cows were homogenous, as determined by Levene’s test (Table 2). The two groups were compared based on milk yield during the first 100 days of lactation using an independent samples *t*-test. On average, AI cows produced 3400.2 kg of milk, whereas cows in the MOET group produced 3555.5 kg, a statistically significant difference favoring the MOET group (*p* < 0.05).

## 4. Discussion

Our study indicates that reproductive technologies may influence culling dynamics, health profiles, and early lactation performance in dairy cows. Reproductive disorders were the predominant reason for culling in both AI- and MOET-derived animals, although the distribution of other health-related causes differed between groups.

The proportion of culling due to reproductive disorders is consistent with earlier reports identifying reproductive inefficiency as the primary cause of involuntary culling in high-producing dairy herds [23]. Impaired fertility prolongs calving intervals, reduces lifetime productivity, and generates substantial economic losses. Although reproductive disorders were the leading culling reason in both groups, MOET-born cows showed a higher proportion (60%) of animals free from such disorders. This pattern suggests that culling decisions may reflect not only the presence of disorders but also their severity, recurrence, or management thresholds, as previously reported [24].

Hoof disorders represented the second most frequent culling reason in AI cows (21.1%) and were slightly less frequent in MOET animals (18.1%). This finding is consistent with studies identifying lameness as a major factor affecting welfare and longevity in dairy herds [25]. As no statistically significant difference in hoof disorder incidence was detected between the groups, the observed differences in culling frequency may reflect management-related factors rather than inherent susceptibility.

Mortality and metabolic diseases were more frequently recorded as culling reasons in MOET cows. Although the overall incidence of metabolic disorders was not higher, cases leading to culling may have been more severe or less responsive to treatment. Similar patterns have been reported in longitudinal studies [26,27,28]. Reproductive disorders were also associated with increased culling risk, consistent with previous findings [7,29].

Udder health also influenced productive lifespan. The lower mastitis incidence observed in MOET-born cows supports previous findings suggesting that genetic background and selection intensity may influence resistance to udder infections [30]. Improved udder health contributes to better animal welfare, reduced veterinary costs, and enhanced milk quality, underlining its economic and biological importance.

Despite apparent health advantages, MOET cows exhibited a significantly higher overall culling risk. This finding suggests an association between embryo-derived origin and factors potentially affecting longevity or productive lifespan. Similar patterns have been reported in high-genetic-merit cows, where superior production traits may coincide with reduced functional longevity [31]. As embryo transfer is typically applied to elite donor lines, offspring may inherit both high production potential and potential vulnerabilities associated with intensive selection, including increased inbreeding or reduced robustness. In addition, higher farmer expectations toward genetically superior cows may lower tolerance for performance deviations, thereby influencing culling decisions.

MOET cows also demonstrated superior milk production during early lactation, likely reflecting the higher genetic merit of donor females. While this is consistent with previous studies [32,33], contrasting results have also been reported [21,34,35], indicating that differences in herd management, genetic background and study design may influence outcomes. The observed pattern supports the well-documented trade-off between productivity and longevity in modern dairy cattle.

A limitation of the current study is that breeding values were not explicitly included in the analysis. Although identical bulls were used for both groups, donor females selected for MOET programs typically represent the upper range of breeding values within the herd. Therefore, part of the observed differences may reflect inherited genetic advantages rather than the technology itself. Consequently, the results should be interpreted with caution, and future studies incorporating genomic or estimated breeding values would allow a more precise separation of genetic and technological effects.

Overall, the findings suggest that reproductive technology is associated with complex and multifactorial effects on health, production and longevity. These results highlight the importance of breeding strategies that balance production traits with functional and health-related characteristics to ensure sustainable herd performance.

## 5. Conclusions

In conclusion, the findings of this study indicate that reproductive technology may be associated with differences in the health profile and survival dynamics of dairy cows. MOET-born animals were associated with a lower incidence of reproductive, metabolic, and mammary disorders and tended to show superior early lactation performance; however, they also exhibited an elevated overall risk of culling. These results highlight the multifactorial nature of culling decisions, in which genetic background, health status, management strategy, and production potential interact. Given that MOET programs are typically applied to genetically superior donor animals, part of the observed differences may also reflect underlying genetic merit rather than the reproductive technology alone. Future research should aim to further disentangle the genetic and environmental determinants underlying these associations and to develop breeding strategies that balance productivity with productive lifetime, longevity and herd sustainability in dairy populations.

## Figures and Tables

**Table 1:** Distribution of culling reasons of culled cows (percentages show the ratio of culled cows to the total number of cows per group).

Reproduction Techniques	Culling Reason							Total Number of Culled Cows	Total Number of Cows
	1	2	3	4	5	6	7		
AI	54 ^a^ (3.5%)	86 (5.6%)	151 (9.8%)	288 (18.7%)	68 (4.4%)	68 (4.4%)	2 (0.1%)	717 (46.4%)	1544 (100%)
MOET	18 ^b^ (7.5%)	21 (8.8%)	21 (8.8%)	43 (18.0%)	3 (1.3%)	10 (4.2%)	0 (0.0%)	116 (48.5%)	239 (100%)

1: metabolic diseases, 2: death, 3: hoof disorders, 4: reproductive disorders, 5: accident, 6: udder-related problems, and 7: other. ^a,b^: the different letters show significant differences between groups within categories (*p* < 0.05).

**Table 2:** Milk production (kg) over first 100 days of lactation (mean ± std. err.).

Reproduction Techniques	Number of Cows	Milk Production (100 Days) (kg)
AI	1544	3400.2 ± 14.42 ^b^
MOET	239	3555.5 ± 38.87 ^a^

^a,b^: the different letters show significant differences (*p* < 0.05).

## Data Availability

The original contributions presented in this study are included in this article. Further inquiries can be directed to the corresponding author(s).

## Abbreviations

The following abbreviations were used in this manuscript:

AI	Artificial insemination
MOET	Multiple ovulation embryo transfer
ET	Embryo transfer
ART	Assisted-reproduction technologies
GS	Genomic selection
